# Using Eye Tracking to Measure Video Game–Assisted Therapy for Improved Visual Outcomes in Pediatric Strabismus: Randomized Control Trial

**DOI:** 10.2196/66538

**Published:** 2026-05-25

**Authors:** Ahmad F Klaib

**Affiliations:** 1 Information Systems Department, Faculty of Information Technology and Computer Sciences, Yarmouk University, Shafiq Irshidat Street, P.O. Box 566, Irbid, 21163, Jordan; 2 Department of Computer Engineering, College of Engineering, Al Yamamah University, Al Khobar, Saudi Arabia

**Keywords:** eye tracking techniques, pediatric strabismus, video game–assisted therapy, Tobii eye tracker, visual outcomes

## Abstract

**Background:**

Strabismus, commonly known as crossed eyes, is a condition characterized by the misalignment of the eyes, leading to impaired binocular vision and depth perception. Traditional management often involves occlusion therapy, which can be hindered by poor compliance. Video game therapy has emerged as a promising adjunct to traditional treatments, potentially improving compliance and directly stimulating visual and cognitive functions.

**Objective:**

This study compares the effectiveness of traditional occlusion therapy with and without the addition of video game therapy in improving visual outcomes among children with strabismus.

**Methods:**

This randomized, controlled clinical trial included 30 children aged 5 to 10 years diagnosed with various types of strabismus. Participants were randomly assigned to 2 groups: the control group (eye patching alone) and the treatment group (eye patching combined with video game therapy). The intervention lasted 3 months, with data collected at baseline and after the treatment. Primary outcomes included visual acuity, angle of deviation, and stereoacuity, while fixation stability was measured using the Tobii eye tracker. Data were analyzed using paired 2-tailed *t* tests, independent 2-tailed *t* tests, Mann-Whitney *U* tests, and multivariate analysis of variance, with a significance level set at *P*<.05.

**Results:**

The treatment group showed significant improvements across all measured outcomes. Visual acuity improved significantly in both the amblyopic (*P*<.001) and fellow or nonamblyopic eyes (*P*<.001). The angle of deviation decreased (*P*<.001), and stereoacuity improved (*P*<.001). Fixation stability, as measured by the eye tracker, also showed significant improvement and was higher in the treatment group (mean 2.87, SD 0.56 seconds) compared with the control group (mean 1.88, SD 0.42 seconds; *P*<.001). The control group, which received only eye patching, also exhibited significant improvements, though to a lesser extent. Posttreatment visual acuity in the amblyopic eye was significantly better in the treatment group compared to the control group (*P*<.001), with similar results for the fellow or nonamblyopic eye (*P*<.001). Stereoacuity also showed superior improvement in the treatment group (*P*=.01). Fixation stability was significantly better in the treatment group (*P*<.001), and multivariate analysis of variance indicated a highly significant overall difference between the 2 groups across multiple outcomes (*P*<.001).

**Conclusions:**

The combination of traditional eye patching with video game therapy significantly enhances treatment outcomes in children with strabismus, particularly in improving visual acuity, stereoacuity, and fixation stability. While both interventions effectively reduce the angle of deviation, video game therapy offers additional benefits by engaging in and improving the functional aspects of vision. These findings suggest that incorporating video game therapy into traditional treatment regimens could lead to more effective and engaging interventions for pediatric strabismus, potentially improving compliance and long-term outcomes. Future research should focus on understanding the underlying mechanisms and optimizing therapy for diverse patient populations.

## Introduction

### Background

Strabismus, commonly known as “a binocular ocular coordination disorder characterized by the misalignment of the eyes, where one or both eyes deviate from the intended point of fixation. This misalignment can interfere with binocular vision and depth perception but does not necessarily lead to vision loss unless complicated by additional conditions” [1]. It is a prevalent pediatric ocular disorder, affecting approximately 1.93% of children worldwide [2]. Early diagnosis and treatment are crucial to prevent the development of amblyopia, commonly referred to as lazy eye, a condition where prolonged misalignment leads to visual deprivation, and the brain begins to suppress the image from the misaligned eye [3,4].

Traditional management of strabismus typically involves the use of occlusion therapy, where an eye patch is placed over the stronger eye to encourage the weaker eye to work harder, thereby improving its visual function [5]. However, the success of occlusion therapy is often limited by poor compliance, especially among children who find the treatment uncomfortable or socially stigmatizing [6]. As a result, there has been growing interest in integrating more engaging, child-friendly interventions with traditional therapies. One such intervention is video game therapy, which holds the potential not only to enhance compliance but also to directly stimulate visual and cognitive functions through immersive, interactive gameplay [7].

This study aims to compare the effectiveness of traditional occlusion therapy with and without the addition of video game therapy in improving visual outcomes among children with strabismus. By using both therapeutic modalities, this research seeks to provide evidence for a more effective, engaging, and sustainable approach to treating strabismus.

### Strabismus and Its Impact

Strabismus is a multifactorial condition characterized by the misalignment of the eyes, which prevents them from focusing on the same point simultaneously [8]. This misalignment can be constant or intermittent and may involve 1 or both eyes deviating inward (esotropia), outward (exotropia), upward (hypertropia), or downward (hypotropia) [9,10]. Amblyopia is defined as “a neurodevelopmental vision disorder characterized by a reduction of best-corrected visual acuity in one or both eyes, resulting from abnormal visual experience during the critical period of visual development. It arises due to disrupted binocular interaction (e.g., strabismus, anisometropia, or visual deprivation) and is not simply a suppression of visual input.” Although strabismus and amblyopia can coexist, they are distinct conditions: strabismus reflects ocular misalignment, while amblyopia reflects impaired visual development [8].

The development of strabismus is influenced by a combination of genetic and environmental factors. Genetic predisposition plays a significant role, with studies indicating a higher prevalence among individuals with a family history of the condition [11]. Environmental factors, such as visual stress or neurological disorders, can also contribute to the onset of strabismus [12]. The condition poses significant challenges to the affected individuals, particularly children, as it can affect their social interactions, academic performance, and overall quality of life [13].

### Traditional Treatments for Strabismus

The primary goals of strabismus treatment are to restore proper eye alignment, improve binocular vision, and prevent amblyopia. Traditional treatment modalities include corrective glasses, prism lenses, vision therapy, and, in some cases, surgical intervention [14]. Among these, occlusion therapy, or patching, remains a cornerstone of treatment, particularly for children with amblyopia [14]. Patching works by forcing the brain to use the weaker eye by occluding the stronger one, thus stimulating visual development in the affected eye [15].

Despite its effectiveness, occlusion therapy is often associated with challenges, particularly regarding compliance. Studies have shown that children frequently resist wearing eye patches due to discomfort or social embarrassment, leading to suboptimal outcomes [16]. Additionally, patching alone may not adequately address the underlying binocular dysfunction, highlighting the need for adjunctive therapies that can enhance the overall treatment effect [17].

### The Emergence of Video Game Therapy

In recent years, video game therapy has emerged as a promising adjunct to traditional strabismus treatments. Video games designed for therapeutic purposes can be customized to engage specific visual and cognitive functions, such as eye-hand coordination, visual tracking, and depth perception [7]. These games are often interactive and visually stimulating, making them particularly appealing to children and potentially improving treatment adherence [7].

The effectiveness of video game therapy in improving visual outcomes has been demonstrated in several studies. For instance, Birch et al [18] conducted a study where children with amblyopia played a custom-designed video game during occlusion therapy, resulting in significant improvements in visual acuity and binocular function compared to traditional patching alone. Similarly, Vedamurthy et al [19] used a dichoptic video game to treat adult amblyopia, reporting improvements in visual acuity and contrast sensitivity. However, these studies did not incorporate eye tracking technology, leaving a gap in understanding the precise mechanisms through which video game therapy exerts its therapeutic effects.

### The Role of Eye Tracking in Strabismus Treatment

Eye tracking technology has evolved significantly, offering precise and noninvasive methods to monitor and analyze eye movements. It involves measuring the position and movement of the eyes, often using advanced algorithms to track the gaze direction, saccades, and fixations. There are several prominent techniques used in eye tracking, including scleral search coil, infrared oculography, electrooculography, and video oculography [20]. Among these, video oculography, which uses video cameras to record eye movements and estimate gaze direction, is particularly common due to its noninvasive nature and ability to track eye movements accurately in real time [20]. Recent advancements, such as the integration of machine learning and Internet of Things, have further enhanced the capabilities of eye tracking systems, allowing for real-time data processing and the automation of gaze detection [20]. These technological improvements enable more sophisticated applications, including medical diagnosis and human-computer interaction [20]. By providing accurate gaze data, eye tracking facilitates a deeper understanding of visual attention and cognitive processes in various fields, from marketing to health care.

Eye tracking technology provides a noninvasive means of assessing visual function and ocular motility with high accuracy [21]. The Tobii eye tracker, in particular, allows for the detailed analysis of fixation stability, saccades, and smooth pursuit movements, which are critical in understanding how the eyes coordinate and maintain focus on a target [22]. This is particularly relevant for patients with strabismus, where poor fixation stability is common, leading to difficulties in maintaining binocular vision and increasing the risk of amblyopia [23].

In strabismus treatment, assessing fixation stability is crucial because it reflects the ability of the eyes to maintain a steady gaze on a single point [24]. By using eye trackers, clinicians can objectively measure improvements in eye stability following treatment, providing a clear metric for treatment success [25]. Moreover, the integration of eye tracking in therapy allows for real-time feedback during visual exercises, which can enhance the effectiveness of treatments like video game therapy by ensuring that the exercises target specific deficiencies in eye movement and coordination [25].

### The Integration of Eye Tracking and Video Game Therapy

The combination of video game therapy with eye tracking technology represents a significant advancement in the treatment of strabismus. While video games alone have been shown to improve visual outcomes, integrating eye tracking provides a deeper understanding of how these improvements occur. Eye tracking allows for the monitoring of fixation stability and eye movement patterns during gameplay, offering real-time feedback that can be used to tailor the therapeutic experience to the specific needs of the patient [26].

Furthermore, eye tracking can help identify which aspects of the game are most effective in improving visual function, enabling the development of more targeted and efficient therapies. By capturing detailed eye movement data, researchers and clinicians can better understand the relationship between gameplay, eye movement control, and overall visual improvement.

This study aims to build on previous research by combining video game therapy with eye tracking to enhance the treatment of strabismus. By doing so, it seeks to provide a more comprehensive treatment approach that not only improves visual acuity and binocular vision but also enhances eye movement control, leading to more robust and sustained treatment outcomes.

## Methods

### Ethical Considerations

Ethics approval for the study was obtained from the institutional review board of Yarmouk University (reference IRB/2024/371), and informed consent was obtained from the parents or guardians of all participants. The study data were fully deidentified and anonymized prior to analysis, ensuring the privacy and confidentiality of all participants. The study was elective, and no monetary or material compensation was provided to participants for their involvement in the study.

### Study Design

Demographic information, including age and sex, type and laterality of strabismus, and history of eye patching treatment, was collected using a structured patient-facing questionnaire completed by the child’s parent or guardian. The questionnaire (Multimedia Appendix 1) was designed in the Arabic language and included closed-ended questions to ensure consistency of responses. Participation was voluntary, and all data were collected anonymously (Multimedia Appendix 2). All information collected was used for research purposes only and treated with strict confidentiality. The identity of the child or any personal data was not disclosed at any stage of the study.

This study is a randomized, controlled clinical trial designed to compare the effectiveness of 2 treatment modalities for strabismus in children: eye patching combined with video game therapy versus eye patching alone. The study was conducted over a period of 3 months and included children aged 5 to 10 years who were diagnosed with various types of strabismus, including esotropia, exotropia, and hypertropia. Data were collected at 2 time points: before the initiation of treatment (baseline) and after the completion of the 3-month treatment period. To assess the impact of the interventions, the Tobii eye tracker was used to measure fixation stability, providing precise and objective data on the consistency and duration of gaze fixation at both the baseline and final assessment.

### Participants

The target population for this study was children aged 5 to 10 years diagnosed with strabismus. Participants were recruited from the outpatient ophthalmology clinic at Al-Basheer Hospital, Amman, Jordan, between January and August 2024. Inclusion criteria were as follows: age between 5 and 10 years, diagnosis of strabismus confirmed by an ophthalmologist, no prior history of surgical intervention for strabismus, and willingness to adhere to the treatment regimen and attend follow-up visits. Exclusion criteria included the presence of any neurological or systemic conditions that could affect vision, previous treatment with occlusion therapy for more than 3 months, and any contraindication to the use of eye patches or video game therapy.

Although participants with more than 3 months of previous occlusion therapy were excluded, 10 children had a history of short-term treatment prior to enrollment. These treatments included brief periods of patching and/or optical correction with glasses. Detailed data on the duration and adherence of these prior treatments were not consistently available.

A total of 30 participants were enrolled in the study, with 15 children randomly assigned to the control group (eye patching alone) and 15 to the treatment group (eye patching combined with video game therapy). Randomization was performed using a computer-generated random sequence.

### Tobii Eye Tracker

The Tobii eye tracker (Tobii Pro X3-120) was securely mounted on a dedicated stand positioned directly in front of the participant, approximately 60 cm from the child’s eyes, at eye level. This setup ensured consistent and accurate tracking of the participant’s eye movements. Each child participates in the calibration process that is provided on the screen to ensure that the system accurately tracks their gaze across different areas of the screen. The eye tracker continuously monitors whether the child is looking at the screen or not by capturing real-time data on the duration and consistency of the gaze fixation. The eye tracker during sessions logs the track gaze position and detects fixations and timestamps to measure engagement, while excluding periods where gaze deviates from the screen for extended durations. The data collected by the eye tracker were automatically recorded and analyzed using the Tobii Pro Lab software, providing detailed insights into the child’s fixation stability over time.

### Intervention

The intervention group received a combination of eye patching and video game therapy. Participants in this group were instructed to wear an eye patch over the nonamblyopic eye for a total of 2 hours per day, during which they played a specifically designed video game aimed at improving visual acuity and binocular vision. The video game sessions lasted 30 minutes each day, 5 days a week, for a total of 3 months. The video game used in this study was developed to encourage visual tracking, eye-hand coordination, and depth perception, with levels of difficulty progressively increasing over time to match the child’s improving visual capabilities.

The control group received standard occlusion therapy, in which the nonamblyopic eye was patched for 2 hours per day without any additional visual exercises or video game therapy. Parents of children in both groups were provided with detailed instructions on the proper use of the eye patch and were asked to maintain a daily log to record the duration of patching. As part of the routine clinical instructions, the attending ophthalmologist advised the children to engage in near-vision activities such as reading, writing, or drawing during the patching period as a form of passive visual therapy. However, adherence to these suggested activities could not be reliably confirmed, as children may not consistently perform them at home. In contrast, the video game–based therapy provided a structured and motivating experience that encouraged regular visual engagement and compliance with the therapy regimen.

All participants who required refractive correction wore their prescribed glasses during both the ophthalmologic examinations and the video game therapy sessions to ensure optimal visual performance and standardization of testing conditions.

### Clinical Evaluation

Ocular motility was assessed by a pediatric ophthalmologist through a standard 9-gaze examination. Motility was considered normal when full, symmetric ocular movements were observed in all gaze positions without limitation or overaction. It was classified as abnormal if any limitation, restriction, or overaction of extraocular muscles was noted in 1 or more directions of gaze.

### Game Development

#### Game 1: Object Manipulation Based on Strabismus Type

**Figure 1.**
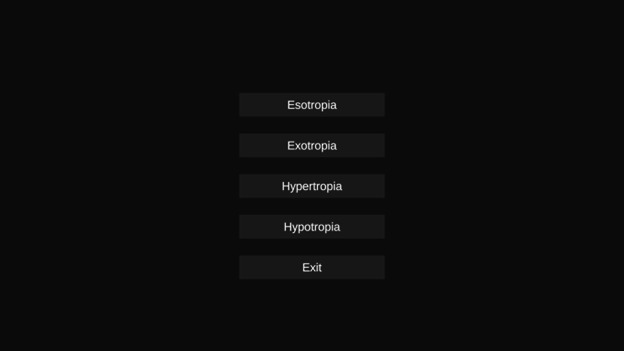
Object manipulation game’s user interface: the interface allows the physician to select the type of strabismus, esotropia, exotropia, hypertropia, or hypotropia, based on the child’s condition. Accordingly, the game’s objects will appear and move in different ways related to their strabismus type.

**Figure 2.**
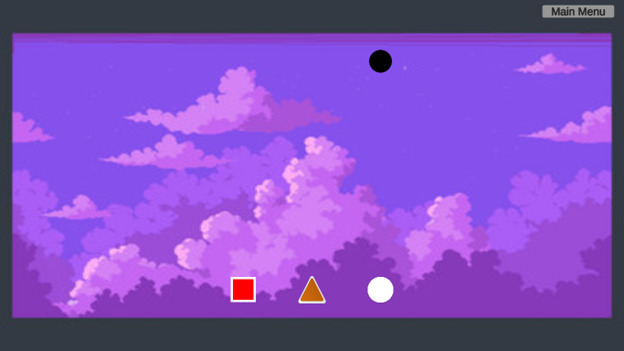
Visual of the object manipulation game’s background: this figure depicts the game’s background environment with objects like squares, triangles, and circles. These objects serve as targets for the children to move and track according to their strabismus-related movement, reinforcing the connection between gameplay and visual improvement.

The first game developed for this study involves manipulating objects based on the specific type of strabismus diagnosed by the physician. Before the game begins, the physician selects the type of strabismus, esotropia, exotropia, hypertropia, or hypotropia, based on the child’s condition, after which the Tobii eye tracker is activated to monitor eye movements during the gameplay as shown in Figure 1. In this game, the child is presented with a series of objects that they must hold and move to a target location. The game progresses through 3 levels, with each level increasing the number of objects to be manipulated, thereby enhancing the child’s ability to control their eye movements and improve visual acuity and coordination. For esotropia, the objects initially appear on the outer edges of the screen and must be moved toward the center, mimicking the inward eye deviation. In the case of exotropia, the objects start in the center and are moved outward toward the edges, reflecting the outward eye deviation. For hypertropia, objects must be moved from a lower position to a higher target, representing the upward deviation of 1 eye, while for hypotropia, the objects are moved from a higher position to a lower target, simulating the downward eye deviation. The progression of the game, with increasing levels of difficulty, helps the children engage both eyes in tasks that challenge their binocular vision, hand-eye coordination, and depth perception. Figure 2 represents a visual of the game’s background, with objects like squares, triangles, and circles. These objects serve as targets for the children to move and track according to their strabismus-related movement, reinforcing the connection between gameplay and visual improvement.

**Figure 3.**
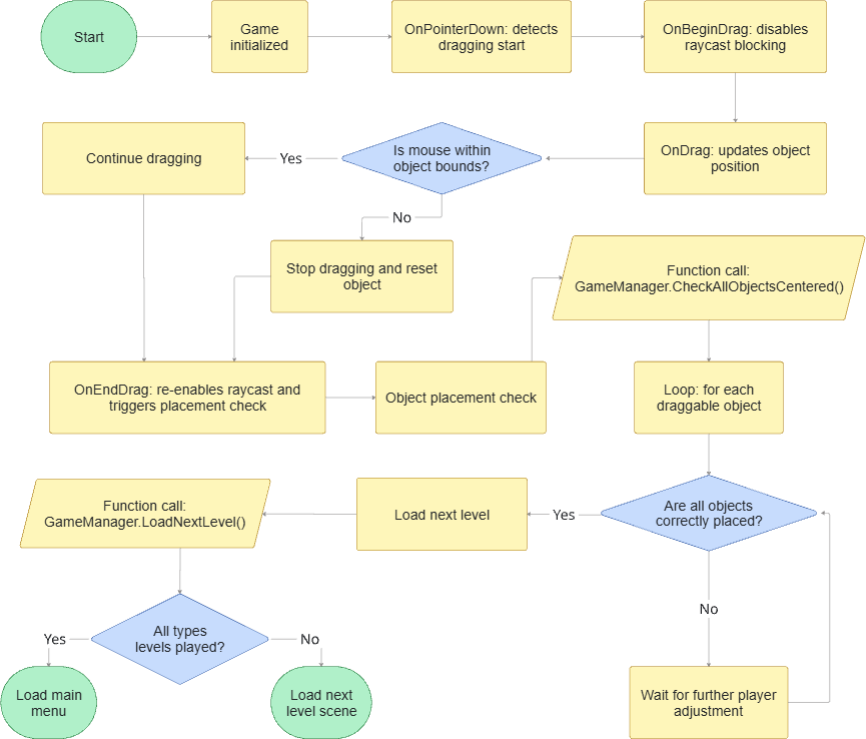
Object manipulation game’s flowchart: the flowchart presents the game’s step-by-step process, begins with game initialization and proceeds through drag-and-drop mechanics, including event handling for dragging, object position updates, and placement checks. Upon successful object alignment, the game advances to the next level or returns to the main menu once all levels are completed.

This game is a Unity-based interactive game developed in C# and features 3 levels for each of 4 types of eye movements: horizontal (exotropia and esotropia) and vertical (hypertropia and hypotropia). Dragging and dropping mechanics are implemented using Unity’s event interfaces (IPointerDownHandler, IBeginDragHandler, IDragHandler, and IEndDragHandler), enabling precise control over object movement. The DragDrop script restricts object movement along predefined paths, either horizontally or vertically, based on the scene configuration. The GameManager script monitors the alignment of objects with their designated slots using vector calculations (RectTransform anchored positions) and automatically loads the next level when all objects are correctly placed. Scene transitions are handled dynamically by checking scene names and indices to determine when to end the game and return to the main menu. This structured approach allows for controlled tracking of eye movements and data collection, providing valuable insights for medical research. The workflow is shown in Figure 3. The workflow begins with game initialization and proceeds through the drag-and-drop mechanics, including event handling for dragging, object position updates, and placement checks. Upon successful object alignment, the game advances to the next level or returns to the main menu once all levels are completed.

#### Game 2: Bug Tracking Based on Strabismus Type

As the first game in this Unity-based project, the experience begins with a transition to the main menu after the player selects “Start Game.” From there, the player navigates to the “Select Level” option, where they choose the type of challenge, such as hypertropia. Upon selecting a challenge, the enemy spawner is activated, and its spawn method is invoked to generate enemies. The next step involves calculating the enemies’ spawn positions relative to the player’s screen size through the “Spawn Position Around Camera” function.

The core of enemy interaction revolves around player actions. If an enemy is clicked, it is killed, and its color changes to indicate its defeat. If the enemy is not clicked, it proceeds to move toward the tower. Upon reaching the tower, an “Eating Cake” animation is triggered, and the cake’s appearance visibly changes as it is consumed.

**Figure 4.**
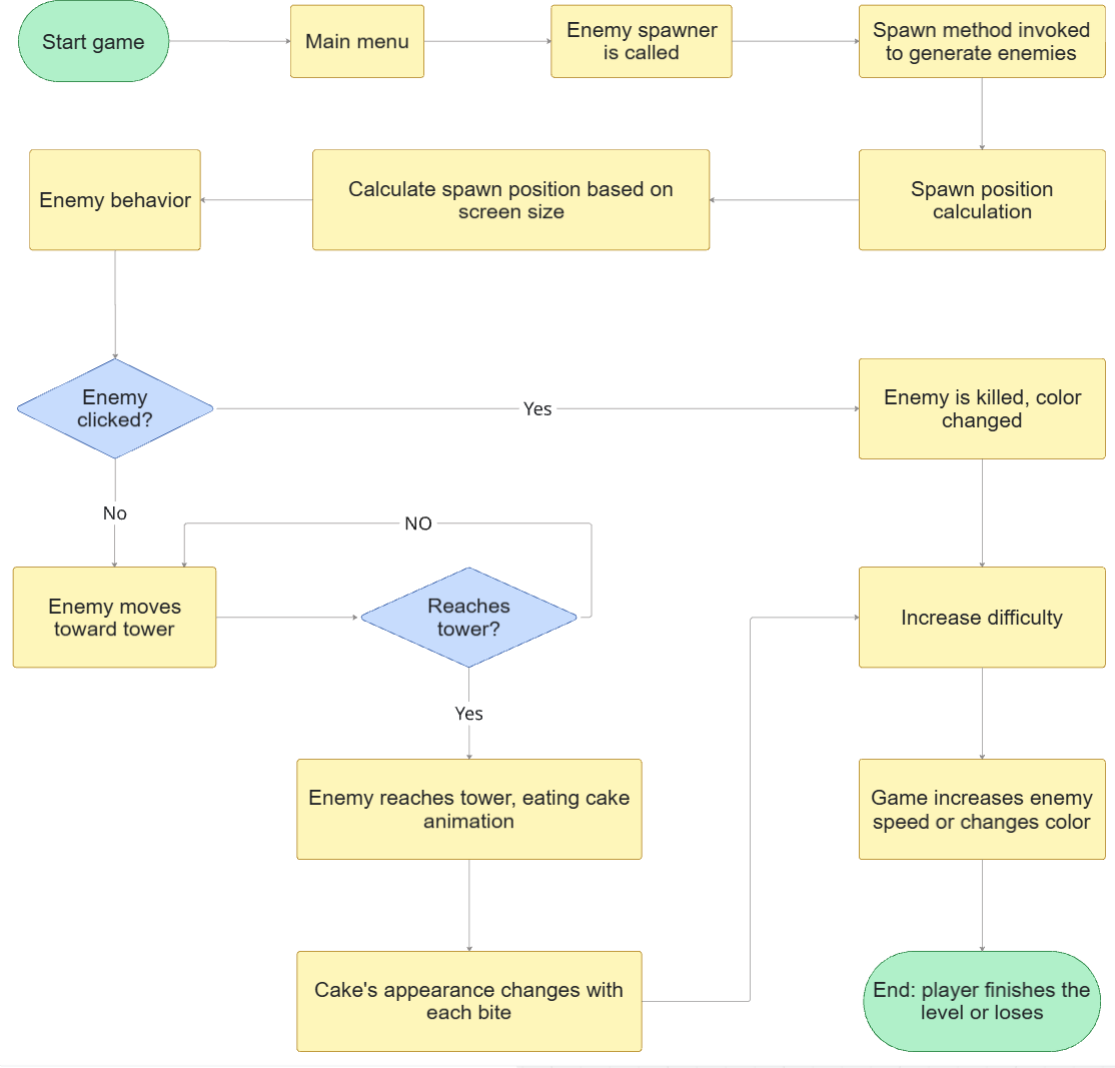
Bug tracking game’s flowchart: the flowchart presents the game’s step-by-step process, begins with a transition to the main menu, selecting the game’s level to choose the type of challenge, activating the enemy spawner, and generating enemies with an aim of killing them to reach the tower and eating cake. The game ends when the player either successfully completes the level or loses the challenge.

Throughout the gameplay, difficulty is dynamically increased by periodically accelerating enemy speed or altering their color. The game concludes when the player either successfully completes the level or loses the challenge. This process is shown in Figure 4.

**Figure 5.**
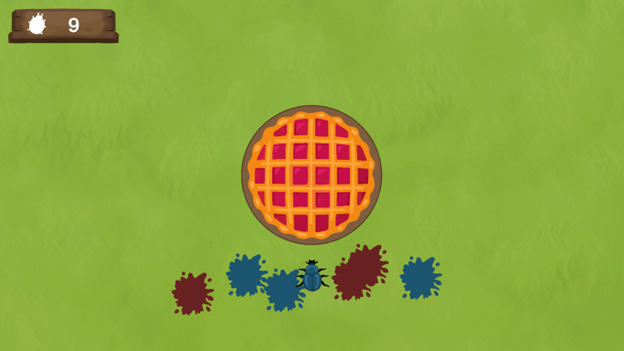
Visual of the bug tracking game’s background: this figure depicts the game’s background environment with objects like bugs and enemies. The bug moves in a direction depending on the strabismus type to kill enemies and eat the cake object.

The second game, shown in Figure 5, involves a bug that the child must track and move in a manner dependent on the type of strabismus they have. As in the first game, the physician selects the appropriate type of strabismus using the initial menu, and once selected, the Tobii eye tracker monitors how well the child can follow the bug’s movement on the screen. The bug moves in specific directions based on the type of strabismus: for esotropia, the bug moves inward from the periphery of the screen toward the center, mimicking the inward deviation of the eye; for exotropia, the bug starts in the center and moves outward toward the edges, representing the outward deviation; for hypertropia, the bug moves upward, simulating the upward eye deviation; and for hypotropia, the bug moves downward, corresponding to the downward deviation of the eye. By engaging in this activity, the child strengthens their eye tracking ability and visual fixation, both of which are crucial for effective strabismus treatment.

### Outcomes and Measurements

The primary outcomes of the study were the improvement in visual acuity, reduction in the angle of deviation, and improvement in stereoacuity. Secondary outcomes included ocular motility and fixation stability as measured by an eye tracker.

#### Visual Acuity

Visual acuity was measured for both the amblyopic and fellow or nonamblyopic eyes using a standardized Snellen chart. The results were recorded in logarithm of the minimum angle of resolution (logMAR) units to provide a more precise quantification of visual improvement.

#### Angle of Deviation

The angle of deviation was measured in prism diopters using the alternate cover test with prism neutralization. Measurements were taken at both near (33 cm) and distance (6 m) fixation to assess the alignment of the eyes.

#### Stereoacuity

Stereoacuity was assessed using the Titmus fly test, which measures the finest level of binocular vision that can be detected by the patient. Results were recorded in seconds of arc.

#### Ocular Motility

Ocular motility was evaluated by an ophthalmologist using a standard 9-gaze test to determine the presence of any abnormal eye movements or restrictions in the range of motion.

#### Fixation Stability

Fixation stability was measured using a Tobii eye tracker (Tobii Pro X3-120). The eye tracker was securely mounted on a dedicated stand positioned directly in front of the participant, approximately 60 cm from the child’s eyes, at eye level. This setup ensured consistent and accurate tracking of the participant’s eye movements. During the assessment, the child was asked to focus on a central target displayed on a computer screen for a period of 30 seconds. The eye tracker captured real-time data on the duration and consistency of the gaze fixation, with the results expressed in seconds. The data collected by the eye tracker were automatically recorded and analyzed using Tobii Pro Lab software, providing detailed insights into the child’s fixation stability over time.

All outcomes were measured at baseline (day 0) and at the end of the treatment period (day 90). To ensure consistency, the same examiner conducted all assessments at each time point.

### Statistical Analysis

Data were analyzed using the Python programming language on Google Colab. Descriptive statistics were used to summarize the baseline characteristics of the study population, including age, sex, duration of strabismus (which refers to the time since the condition was formally diagnosed), and family history. Continuous variables were expressed as mean and SD, and categorical variables were expressed as frequencies and percentages. The primary analysis compared the change in visual acuity, angle of deviation, stereoacuity, and fixation stability between the 2 groups from baseline to the 3-month follow-up. Paired 2-tailed *t* tests were used to assess within-group changes, and independent 2-tailed *t* tests were used to compare between-group differences. For nonnormally distributed data, the Wilcoxon signed rank test and the Mann-Whitney *U* test were applied. A multivariate analysis of variance was also conducted to assess the overall effect of the treatment across multiple dependent variables simultaneously. The significance level was set at *P*<.05.

## Results

### Baseline Characteristics

**Figure 6.**
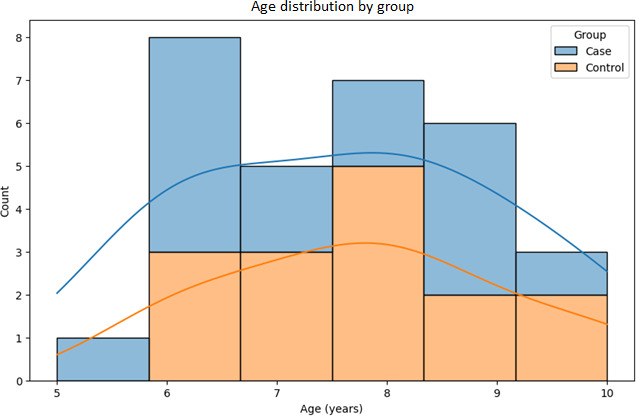
Age distribution of participants in the study by group: this figure displays the age distribution across case and control groups, providing context for the demographic composition of the study.

The study population consisted of 30 children aged 5 to 10 years (mean 7.6, SD 1.43 years) as shown in Figure 6. The mean duration of strabismus among participants was 29.23 (SD 16.62) months, with a range spanning from 2 to 57 months. Sex distribution was slightly skewed toward female participants, who represented 56.7% (17/30) of the cohort, while male participants accounted for 43.3% (13/30). Notably, none of the participants reported a family history of strabismus. Prior to the study, 33.3% (10/30) of the participants had received some form of treatment for strabismus, while the remaining 66.7% (20/30) had not.

### Comparison of the Case and Control Groups

To ensure that the groups are comparable at the baseline, Table 1 summarizes the demographics and clinical characteristics.

**Table 1.** Baseline demographics and clinical characteristics of control and case groups.

Variable	Control group (n=15/30)	Case group (n=15/30)	*P* value^a^	Effect size (Cohen *d*)
Age (years), mean (SD)	6.07 (1.83)	5.47 (1.77)	.36	0.34 (small)
Sex, n (%)			.46	—^b^
Male	6 (20)	8 (26.6)
Female	9 (30)	7 (23.3)

a *P* values were calculated using an independent samples 2-tailed *t* test for continuous variables and a chi-square test for categorical variables.

b Not available.

As shown in Table 1, there is no significant difference between the 2 groups in the baseline characteristics (*P*>.35), which means both groups are well-balanced and supports the validity of treatment comparisons.

### Visual and Motor Function at Baseline

At baseline, the mean visual acuity for the amblyopic eye was 0.59 (SD 0.18) logMAR, and for the fellow or nonamblyopic eye, it was 0.57 (SD 0.16) logMAR. The mean angle of deviation at baseline, measured in prism diopters, was 28.56° (SD 7.60°). Stereoacuity, reflecting binocular vision, had a mean of 613.99 (SD 159.38) seconds of arc. Ocular motility was assessed and classified as either normal or abnormal, with 50% (15/30) of participants exhibiting abnormal motility. Baseline fixation stability, as measured by the eye tracker, averaged 1.06 (SD 0.29) seconds.

### Posttreatment Outcomes

Significant improvements were observed across all outcomes within the treatment group following the intervention. The mean visual acuity improved markedly for both the amblyopic eye (*P*<.001) and the fellow nonamblyopic eye (*P*<.001). The angle of deviation, which indicates ocular alignment, significantly decreased (*P*<.001), suggesting enhanced alignment after the treatment. Stereoacuity also improved substantially, with the mean decreasing to 570.12 (SD 119.54) seconds of arc (*P*<.001), reflecting better binocular vision and depth perception. Fixation stability, as measured by the eye tracker, also showed significant improvement (mean 2.87, SD 0.56 seconds; *P*<.001), indicating enhanced control and consistency in eye movements.

The control group, which received only eye patching, also exhibited significant improvements across all measured outcomes. Visual acuity improved in both the amblyopic eye (*P*<.001) and the fellow or nonamblyopic eye (*P*<.001). The angle of deviation decreased significantly (*P*<.001), and stereoacuity improved, with a mean posttreatment value of 698.74 (SD 145.89) seconds of arc (*P*<.001). Although these improvements were significant, the overall magnitude of change was generally less pronounced compared to the treatment group.

**Figure 7.**
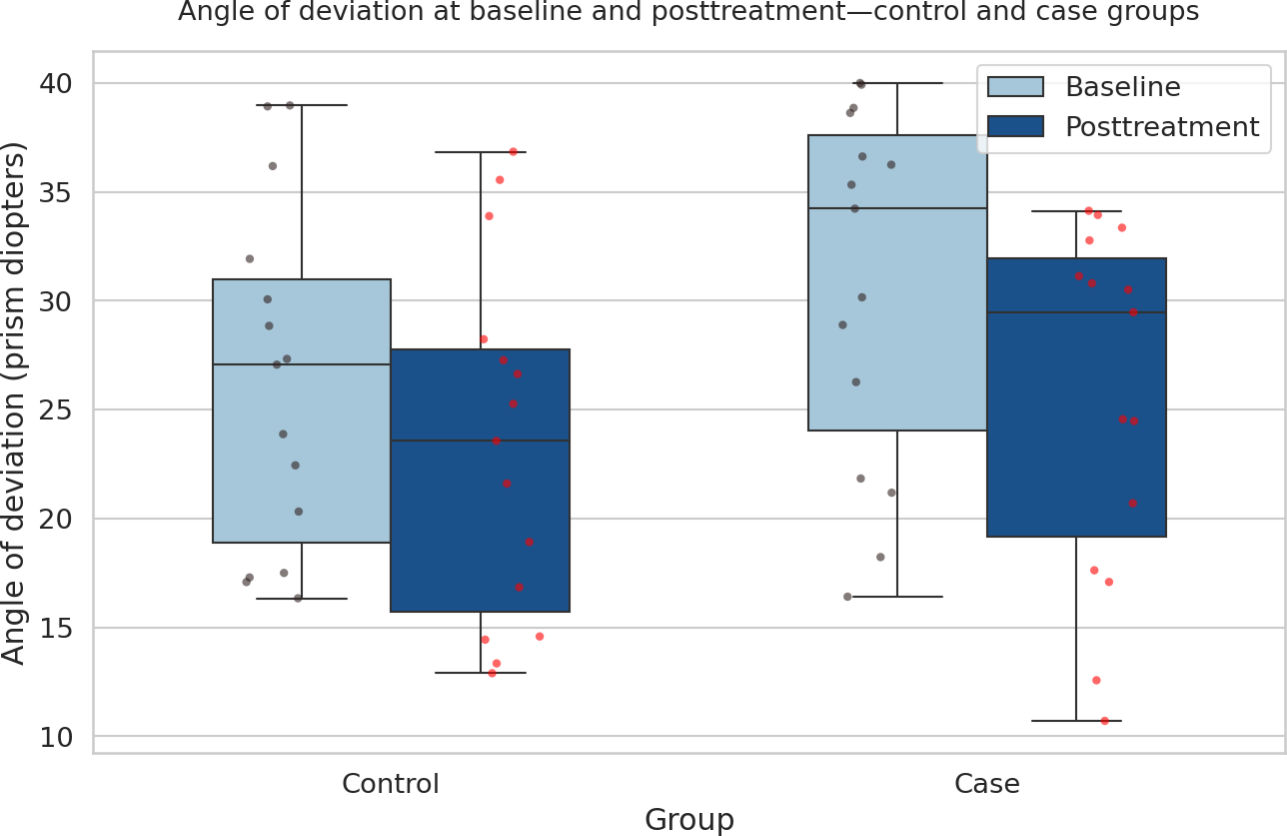
Box plot comparing the angle of deviation (measured in prism diopters) at baseline and after treatment between the control and case groups. The angle of deviation exhibits a wider range in the control group compared to the case group, indicating more variability in the control group’s treatment results. The case group shows a more consistent range of deviation angles, suggesting a potentially more uniform response to the treatment that included video game therapy.

**Figure 8.**
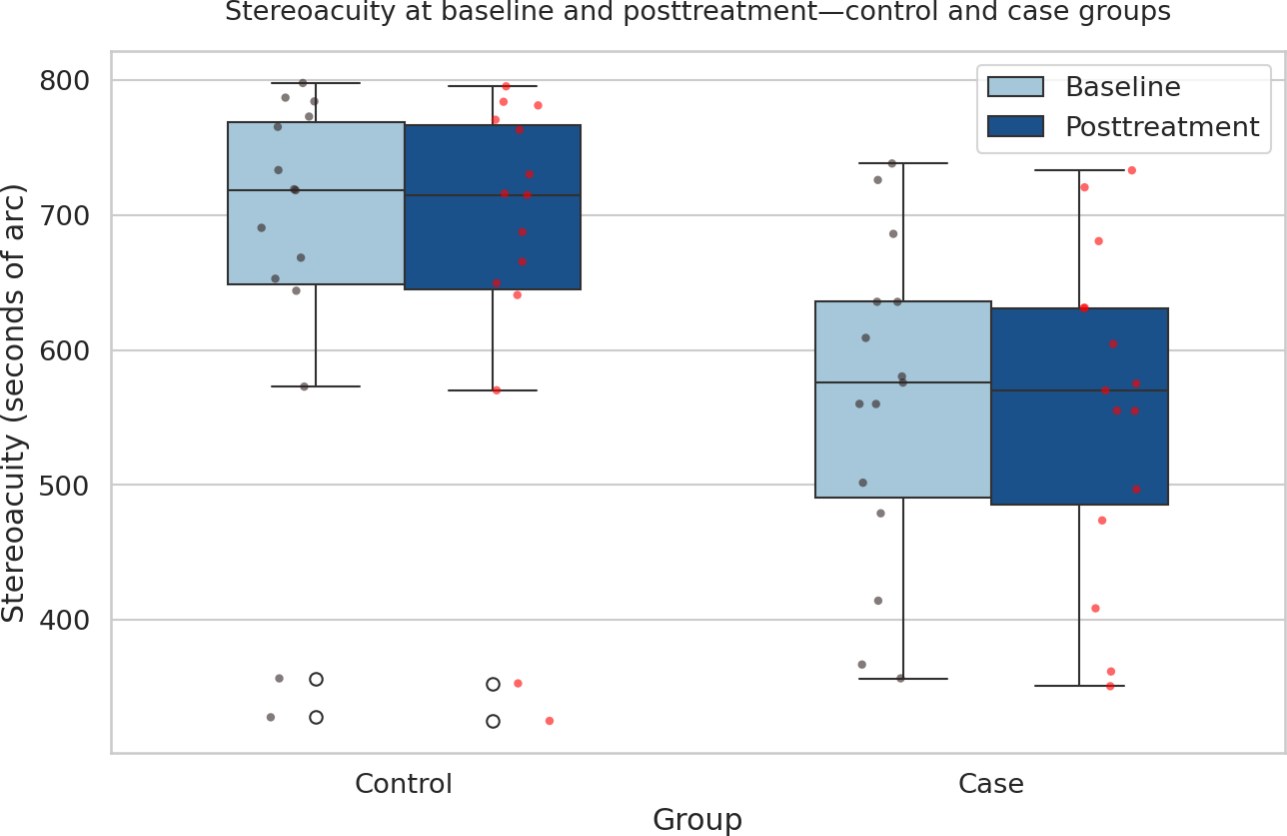
Stereoacuity at baseline and posttreatment for control and case groups. Boxplots display individual participant data (red dots). Lower values indicate better stereoacuity (measured in seconds of arc). Both groups demonstrated posttreatment improvement; however, the treatment (case) group showed a greater reduction in stereoacuity values (mean 570.12, SD 119.54 seconds of arc) compared with the control group (mean 698.74, SD 145.89 seconds of arc), indicating superior enhancement in binocular vision and depth perception (*P=*.01).

**Figure 9.**
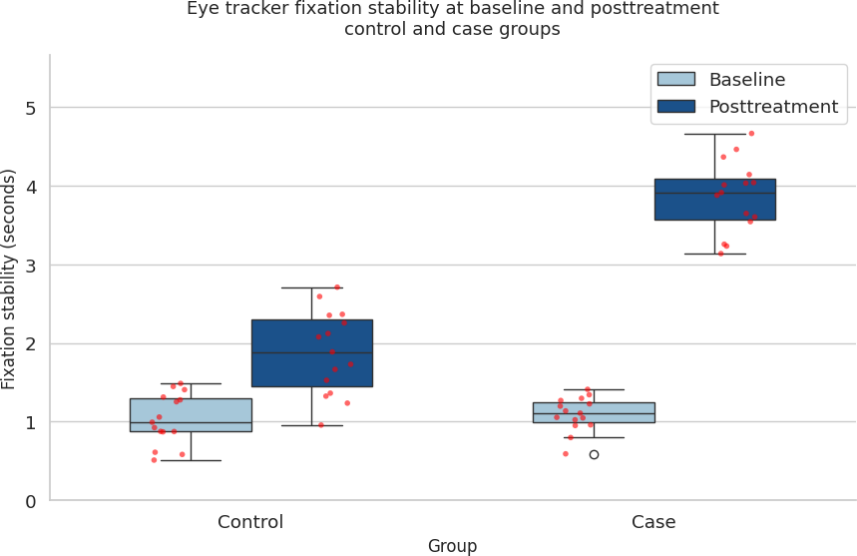
Eye-tracker fixation stability at baseline and posttreatment for control and case groups. Boxplots show individual participant data (red dots). Higher values represent greater fixation stability (measured in seconds). Both groups demonstrated posttreatment improvement; however, the case group exhibited a larger increase (baseline=1.06 seconds; posttreatment=2.87 seconds) than the control group (baseline=1.03 seconds; posttreatment=1.88 seconds), reflecting enhanced ocular motor control following the combined intervention*.*

The comparison between the case and control groups across different metrics reveals notable differences in treatment outcomes. As shown in Figure 7, the angle of deviation (measured in prism diopters) exhibits a wider range in the control group compared to the case group, indicating more variability in the control group’s treatment results. The case group, on the other hand, shows a more consistent range of deviation angles, suggesting a potentially more uniform response to the treatment that included video game therapy. In Figure 8, which compares stereoacuity (measured in seconds of arc), the control group again demonstrates a wider range of outcomes, with several outliers. This wider distribution suggests that the control group experienced more varied improvements in stereoacuity. In contrast, the case group shows a more concentrated distribution of stereoacuity scores, implying a more consistent improvement in their depth perception after treatment. Finally, Figure 9 highlights the differences in fixation stability (measured in seconds) between the groups. Both groups demonstrated posttreatment improvement. The control group shows a narrower range of fixation stability (from 1.03 to 1.88 seconds), indicating a more stable but less varied outcome. The case group, however, displays a wider range (from 1.06 to 2.87 seconds), with more outliers, suggesting that while some individuals in the case group achieved better fixation stability, there was also more variation in the treatment’s effectiveness for this outcome. Overall, these figures suggest that while the control group experienced more variability across all metrics, the case group showed more consistent results, especially in terms of angle of deviation and stereoacuity, possibly due to the addition of video game therapy.

Independent samples 2-tailed *t* tests and the Mann-Whitney *U* test were used to assess whether the treatment group showed superior outcomes compared to the control group, with the choice of test determined by the normality of the data. The analysis revealed that posttreatment visual acuity in the amblyopic eye was significantly better in the treatment group compared to the control group (*P*<.001). This pattern was consistent for the fellow or nonamblyopic eye, where the treatment group again outperformed the control group (*P*<.001), indicating that the treatment had a substantial positive impact on visual acuity in both eyes.

When evaluating the angle of deviation after the treatment, the comparison between the 2 groups did not yield a significant difference (*P*=.45), suggesting that both interventions were similarly effective in improving ocular alignment. However, a different outcome was observed for stereoacuity, where the treatment group showed significantly better improvement than the control group (*P*=.01). This finding indicates that the treatment group achieved superior binocular vision, as reflected in enhanced stereoacuity.

Fixation stability, as measured by the eye tracker, also showed significant improvement in the treatment group compared to the control group (*P*<.001). This result suggests that the combination of eye patching with video game therapy not only improved visual outcomes but also enhanced stability and control of eye movements, further supporting the efficacy of the treatment approach.

To assess the collective impact of the treatment on multiple dependent variables simultaneously, a multivariate analysis of variance was conducted. The results indicated a highly significant overall difference between the control and treatment groups when considering visual acuity (amblyopic and fellow or nonamblyopic eyes), angle of deviation, stereoacuity, and fixation stability together (*P*<.001). This finding highlights the superior efficacy of the treatment group across multiple dimensions of visual function.

In conclusion, the combination of eye patching with video game therapy was more effective than eye patching alone in improving visual acuity, stereoacuity, and fixation stability among children with strabismus. While both interventions were similarly effective in reducing the angle of deviation, the treatment group consistently outperformed the control group in other critical measures of visual function. These findings suggest that incorporating video game therapy into traditional eye patching regimens could enhance treatment outcomes for pediatric patients with strabismus.

## Discussion

### Principal Findings

The results of this study highlight the significant advantages of combining video game therapy with traditional eye patching in the treatment of children with strabismus. By comparing the outcomes of children who received eye patching alone to those who underwent a combined treatment regimen, this study provides compelling evidence that video game therapy can substantially enhance the effectiveness of traditional treatments. The observed improvements in visual acuity, stereoacuity, and fixation stability in the treatment group suggest that the addition of video game therapy offers a multifaceted approach to addressing both the sensory and motor deficits associated with strabismus.

One of the most noteworthy findings of this study is the significant improvement in visual acuity in the treatment group compared to the control group. This result aligns with previous research that has demonstrated the potential of video games to stimulate visual pathways and enhance visual acuity, particularly in children with amblyopia [7,19]. However, our study extends this understanding by incorporating eye tracking technology, which provided a detailed analysis of fixation stability. The improvement in fixation stability observed in the treatment group suggests that video game therapy does not merely enhance visual acuity but also contribute to better control and coordination of eye movements. This dual improvement is likely due to the interactive nature of video games, which require sustained attention and precise eye movements, thereby providing a robust stimulus for the visual system.

Stereoacuity, which reflects the quality of binocular vision, also showed significant improvement in the treatment group, with posttreatment values markedly lower than those observed in the control group. This finding supports the hypothesis that video game therapy can play a crucial role in re-establishing binocular function in children with strabismus. The improvement in stereoacuity is particularly important, as it indicates that the treatment is effective not only in aligning the eyes but also in promoting the integration of visual information from both eyes [27]. This dual benefit contrasts with the traditional approach of patching, which focuses primarily on forcing the brain to use the weaker eye, often at the expense of binocular vision.

The use of eye tracking technology in this study provided a novel dimension to the assessment of treatment outcomes. While previous studies have focused primarily on sensory outcomes, such as visual acuity and stereoacuity [7,19], our study’s use of eye tracking allowed for a detailed analysis of motor outcomes, particularly fixation stability. The significant improvement in fixation stability in the treatment group suggests that video game therapy can enhance both sensory and motor aspects of vision, leading to more comprehensive treatment outcomes. This finding is particularly relevant because poor fixation stability is a common issue in children with strabismus, and it is often associated with an increased risk of amblyopia and poor binocular function.

The comparative effectiveness of traditional patching versus the combined treatment regimen also offers valuable insights. While both treatment modalities were effective in reducing the angle of deviation and improving ocular alignment, the combined treatment was notably more effective in enhancing visual acuity, stereoacuity, and fixation stability. This suggests that while patching remains an essential component of strabismus treatment, video game therapy can significantly enhance its effectiveness by stimulating and improving the functional aspects of vision. The combination of these therapies, therefore, offers a more holistic approach to treating strabismus, addressing both the structural alignment of the eyes and the functional integration of visual information.

These findings have important implications for clinical practice. The integration of video game therapy into traditional patching regimens could improve patient outcomes, particularly in children who may be resistant to patching alone. The interactive and engaging nature of video games has the potential to improve compliance with therapy, which is a significant challenge in traditional treatments. Moreover, the use of eye tracking as part of the treatment protocol offers clinicians a valuable tool for monitoring progress and tailoring therapy to individual patient needs. The detailed eye movement data provided by the eye tracker can help identify specific deficits in ocular motility and fixation stability, allowing for more targeted interventions. This personalized approach to treatment could lead to better long-term outcomes and a lower risk of relapse.

### Limitations and Future Directions

Despite the promising results, this study has several limitations. The sample size was relatively small, and the study duration was limited to 3 months, which may not fully capture the long-term effects of the interventions. Additionally, the study focused on a specific age group (5‐10 years), and the findings may not be generalizable to older children or adults with strabismus. In addition, this study did not address the participants’ insights into their experiences, both objective and subjective engagement, and satisfaction.

In addition, a subset of participants (n=10) had received prior short-term treatment in the form of occlusion therapy and/or optical correction before enrollment. Although this was consistent with the inclusion criteria that excluded longer treatment durations for more than 3 months, variations in previous management and the lack of detailed records regarding adherence may have influenced baseline visual acuity and binocular function. Future research with stratified analysis or exclusion of previously treated participants could help clarify the independent effects of video game–assisted therapy.

Future research should aim to include larger, more diverse populations and longer follow-up periods to assess the sustainability of the treatment outcomes and consider more homogeneous cohorts or stratified subgroup analyses to better isolate the effects of therapy across different clinical categories. Additionally, exploring the use of more advanced video game therapies, possibly incorporating virtual reality or augmented reality, could further enhance the therapeutic potential of this approach. Finally, investigating the neurophysiological mechanisms underlying the improvements observed with video game therapy could provide deeper insights into how these interventions influence visual processing in the brain.

This study focuses exclusively on the development and validation of an eye-tracking system rather than on the medical or disease-specific implications of its use. While the methodology may have future clinical applications, the absence of direct medical outcome analysis represents a limitation. Future work should incorporate multidisciplinary collaboration with clinical experts to evaluate and expand the medical applicability of this technology.

The measurement of fixation stability was based on fixation duration (in seconds) rather than more established ophthalmological parameters such as bivariate contour ellipse area or fixation locus analysis. As such, this approach provides only a simplified assessment of fixation performance. Future studies should incorporate bivariate contour ellipse area and other position-based measures to better characterize fixation stability, particularly in patients with amblyopia where eccentric fixation is common.

### Conclusions

The study demonstrates that combining traditional eye patching with video game therapy significantly enhances treatment outcomes in children with strabismus, particularly by improving visual acuity, reducing stereoacuity values, and increasing fixation stability. While both treatments effectively reduce the angle of deviation, video game therapy offers additional benefits by engaging and improving the functional aspects of vision. The integration of eye tracking technology provides valuable insights into the motor aspects of ocular function, allowing for a more comprehensive assessment and personalized treatment approach.

These findings suggest that incorporating video game therapy into traditional treatment regimens could lead to more effective and engaging interventions for pediatric strabismus, potentially improving compliance and long-term outcomes. Future research should continue to explore this promising approach, with a focus on understanding the underlying mechanisms and optimizing therapy for diverse patient populations.

## Supplementary material

10.2196/66538 Multimedia Appendix 1 Patient-facing questionnaire.

10.2196/66538 Multimedia Appendix 2 Consent form 1.

10.2196/66538 Checklist 1 CONSORT-EHEALTH (V 1.6.1) - Submission/Publication Form

## Abbreviations

logMAR: logarithm of the minimum angle of resolution
