# Supplementary material for: Using Eye Tracking to Measure Video Game–Assisted Therapy for Improved Visual Outcomes in Pediatric Strabismus: Randomized Control Trial
Source: JMIR Serious Games. 2026 May 25;14:e66538. doi: 10.2196/66538 (PMC13200800; doi:10.2196/66538)
Supplement: Multimedia Appendix 1 [file games-v14-e66538-s001.pdf]

Yarmouk University

استبيان لجمع بيانات تتعلق ب مرض حول العين لدى الأطفال

المقدمة:

عزيزي ولي أمر الطفل،

يقوم الباحث الدكتور/ أحمد فضل جميل كليب بدراسة بحثية تهدف إلى فهم التجارب المختلفة للأطفال المصابين بالحول وذلك لغايات إجراء دراسة عن استخدام أدوات تتبع العين لتعزيز علاج مرض حول العين لدى الأطفال. نرجو منكم التكرم بالإجابة بشكل دقيق على هذا الاستبيان لجمع معلومات حول مرض حول العين لدى الأطفال.

جميع المعلومات التي يتم جمعها ستُستخدم لأغراض بحثية فقط، وسيتم التعامل معها بسرية تامة. لن يتم الكشف عن هوية الطفل أو أي بيانات شخصية في أي مرحلة من مراحل الدراسة.

أولاً: المعلومات الأساسية

1. عمر الطفل: ________
2. جنس الطفل: ☐ ذكر ☐ أنثى

ثانيًا: معلومات حول مرض حول العين

3. متى بدأ انحراف العين (الحول) بالظهور لدى طفلك؟

☐منذ الولادة ☐قبل عمر السنتين

☐بين عمر 2–5 سنوات ☐بعد عمر 5 سنوات

4. ما نوع الحول الذي يعاني منه طفلك؟

☐حول داخلي (العين تنحرف للداخل) ☐حول خارجي (العين تنحرف للخارج)

☐حول علوي (العين تنحرف للأعلى) ☐حول سفلي (العين تنحرف للأسفل)

5. أي عين لدى الطفل تتأثر أكثر؟

☐العين اليمنى ☐العين اليسرى

☐كلاهما بنفس الدرجة ☐لست متأكدًا

6. هل تلقى الطفل علاجًا للحول؟ (اختر كل ما ينطبق)

☐النظارات الطبية ☐تغطية العين(Eye Patching)

☐قطرات للعين ☐جراحة

☐علاج بصري ☐لم أتلقَ علاجًا

7. إذا خضع الطفل لعملية جراحية، كم مرة؟

☐مرة واحدة ☐مرتين

☐أكثر من مرتين ☐لا ينطبق

ثالثًا: علاج حول العين باستخدام غطاء العين (Eye Patching)

8. هل سبق لطفلك استخدام غطاء العين كجزء من العلاج؟

☐نعم ☐لا

9. أي عين كان طفلك يغطيها؟

☐العين اليمنى ☐العين اليسرى ☐كلتا العينين (في أوقات مختلفة)

10. كم عدد الساعات في اليوم التي يستخدم فيها طفلك غطاء العين؟

☐أقل من ساعتين ☐2-4 ساعات

☐4-6 ساعات ☐أكثر من 6 ساعات

11. كم المدة التي استمر بها طفلك باستخدام غطاء العين؟

☐أقل من 3 شهور ☐3-6 شهور

☐أكثر من 6 شهور ☐ما زال الطفل يستخدم غطاء العين

12. هل التزم طفلك بتعليمات استخدام غطاء العين؟

☐دائماً ☐أحياناً

☐نادراً ☐لم يلتزم أبداً

13. هل أحب طفلك ارتداء غطاء العين، وإذا لا فلماذا؟

☐نعم أحب إرتداء غطاء العين ☐لا لم يحبه، كان الغطاء غير مريح له

☐لا لم يحبه، كان يشعر بالخجل ☐لا لم يحبه، كان الأطفال يسخرون منه

☐لا لم يحبه، كان الطفل لا يرى به جيداً ☐لا لم يحبه، لأسباب أخرى

14. هل تعتقد أن غطاء العين ساعد طفلك في تحسين الرؤية لديه؟

☐نعم ☐لا ☐ لا أعلم
