# Supplementary material for: Using Eye Tracking to Measure Video Game–Assisted Therapy for Improved Visual Outcomes in Pediatric Strabismus: Randomized Control Trial
Source: JMIR Serious Games. 2026 May 25;14:e66538. doi: 10.2196/66538 (PMC13200800; doi:10.2196/66538)
Supplement: Multimedia Appendix 2 [file games-v14-e66538-s002.pdf]

نموذج مشاركة في استبانة

الكلية: تكنولوجيا المعلومات وعلوم الحاسوب
القسم: قسم نظم المعلومات
التاريخ: 2024/07/15

عنوان البحث: استخدام أدوات تتبع العين لتعزيز علاج مرض حول العين لدى الأطفال

يجري الباحث: د. أحمد فضل جميل كليب من جامعة اليرموك دراسة حول استخدام أدوات تتبع العين لتعزيز علاج مرض حول العين لدى الأطفال .
وتهدف الدراسة الحالية الوصول إلى نتائج تمكن الباحث من تطوير إجراء أو أداة علاجية باستخدام تقنيات تتبع العين لتعزيز علاج مرض حول العين لدى الأطفال.

عزيزي المشارك،

لا يوجد هناك أية مخاطر معروفة تترتب على مشاركتك بهذه الدراسة، كما أنه لا يترتب على مشاركتك أية نفقات أو تبعات مادية.
سيتم استخدام المعلومات التي ستزود الباحث بها من أجل تطوير إجراء أو أداة علاجية باستخدام تقنيات تتبع العين لتعزيز علاج مرض حول العين لدى الأطفال. تتكون هذه الاستبانة من عشرة أسئلة وستكون مدة الإجابة عليها من 5-10 دقائق.

يرجى العلم أن مشاركتك في هذه الدراسة هي طوعية وإنه بإمكانك الامتناع عن إجابة أي سؤال دون الحاجة إلى تقديم أي مبرر ولن يتم أخذ أي معلومات شخصية ومحدده للهوية وإنه سيتم التعامل مع المعلومات في هذه الاستبانة بسرية تامة وفي إطار البحث العلمي.

كما يرجى العلم بأنه تم الحصول على موافقة لجنة أخلاقيات البحث العلمي على الإنسان في جامعة اليرموك لإجراء البحث والتي ستقوم بالاطلاع على البيانات وتدقيقها والتأكد من حفظ سرية المعلومات.

توقيع المشارك:..
التاريخ: / / **20**

في حال وجود أية استفسارات يرجى التواصل مع:
اسم الباحث: د. أحمد فضل جميل كليب **رقم الهاتف:** 0777791914 **البريد الالكتروني:** ahmad.klaib@yu.edu.jo
اسم المشرف على البحث: د. أحمد فضل جميل كليب
اسم الجهة الداعمة ان وجدت: لا يوجد

قامت اللجنة المؤسسية لأخلاقيات البحث العلمي على الإنسان في جامعة اليرموك بمراجعة هذا الطلب لإجراء هذه الدراسة، وتسعد بتلقي أي استفسارات أو ملاحظات حول انتهاك خصوصيتك أو حقوقك في هذه الدراسة من خلال التواصل مع منسق اللجنة المؤسسية على هاتف رقم (+96227211111) فرعي (2083) أو الكتابة على عنوان البريد الإلكتروني التالي IRB@yu.edu.jo
